# Cancer Etiology: A Metabolic Disease Originating from Life's Major Evolutionary Transition?

**DOI:** 10.1155/2019/7831952

**Published:** 2019-10-08

**Authors:** B. Poljsak, V. Kovac, R. Dahmane, T. Levec, A. Starc

**Affiliations:** ^1^Faculty of Health Sciences, University of Ljubljana, Laboratory of Oxidative Stress Research, Ljubljana, Slovenia; ^2^Faculty of Health Sciences, University of Ljubljana, Chair of Biomedicine in Health Care, Ljubljana, Slovenia; ^3^Faculty of Health Sciences, University of Ljubljana, Chair of Public Health, Ljubljana, Slovenia

## Abstract

A clear understanding of the origins of cancer is the basis of successful strategies for effective cancer prevention and management. The origin of cancer at the molecular and cellular levels is not well understood. Is the primary cause of the origin of cancer the genomic instability or impaired energy metabolism? An attempt was made to present cancer etiology originating from life's major evolutionary transition. The first evolutionary transition went from simple to complex cells when eukaryotic cells with glycolytic energy production merged with the oxidative mitochondrion (The Endosymbiosis Theory first proposed by Lynn Margulis in the 1960s). The second transition went from single-celled to multicellular organisms once the cells obtained mitochondria, which enabled them to obtain a higher amount of energy. Evidence will be presented that these two transitions, as well as the decline of NAD+ and ATP levels, are the root of cancer diseases. Restoring redox homeostasis and reactivation of mitochondrial oxidative metabolism are important factors in cancer prevention.

## 1. Introduction

Could cancer causation be interpreted as an allegory not to the damaged hardware (damaged genetic material caused by chance mutation) but to an incorrect function of a software (a metabolic program)? Do we thence use wrong approaches to treat the cancer disease with chemotherapy and radiation therapy, which are aimed at destroying the hardware (killing cells), instead of a more sophisticated approach aimed at reprogramming the software inside the cells in order to restore the normal mitochondrial function and metabolism?

There are carcinogenic and tumorigenic cells with zero mutations [1], and there are many somatic mutations in cancer-driver genes in healthy tissue, which does not become a cancer [2], with so-called driver mutations [3]. Furthermore, experiments on the nucleus and mitochondrial transfer revealed that tumorigenic phenotype is upgraded when tumor mitochondria are transferred to a normal cell cytoplasm and vice versa. This can be illustrated by the transplantation of noncancerous mitochondria which can inhibit tumor properties of metastatic cells [4–9]. Additionally, tumorigenesis may be suppressed by normal mitochondrial function [10–12], and metabolic enzymes of the Krebs cycle have been recognized as oncosuppressors [13].

Both abnormalities in tumor suppressor genes (antioncogene acting to inhibit cell proliferation and tumor development) and oncogenes can be caused by impaired mitochondrial function [14]. Aerobic glycolysis of tumors is in some measure displayed by activation of oncogenes or absence of tumor suppressors, which are then additionally intensified by stabilization of the hypoxia-inducible factor (HIF) [15], which encodes for all of the glycolytic enzymes. It seems that fully operating mitochondria regulate apoptosis by releasing cytochrome c [16] and suppressing genes of cancer-like metabolism, which have been conserved from 500,000 million years ago and persist in cells of multicellular organisms. Such a program, which enables the development of cancer, preexists in genes in the nucleus from the season of low O_2_ atmosphere and single-celled life. Namely, cancer cells shift their metabolism toward glycolysis, a strategy that allows for their survival when oxygen is limited [17], and consequently increase the availability of biosynthetic intermediates needed for cellular growth and proliferation [18]. Du [19] proposed a hypothesis that “the survival style of cancer cells was the reevolution from eukaryotic to prokaryotic cells by the alteration of energy metabolism.” A human body is a sum of colonies of cells and their mitochondria. The cells composing the human body are similar to single-celled eukaryotes (existing 500,000 million years ago) although human cells can no longer survive on their own and generally do not use the primitive source of energy, e.g., substrate-level phosphorylation, to produce ATP. The first life emerged on Earth around 3.5 billion years ago, when the early biosphere was more reduced. The increased amounts of dioxygen (O_2_) emerged approximately 2.4 billion years ago when cyanobacteria, as a product of oxygenic photosynthesis, triggered the “Great Oxidation Event” [20]. Due to the elevated O_2_ in the atmosphere, methods of mitigating its toxicity inside cells had to evolve [21], and the existing metabolic pathways had to be reshaped in early aerobic organisms, which adapted to use O_2_ as a high-potential redox couple. Multicellular life appeared more than a billion-and-a-half years ago, and the Cambrian explosion (somewhere around 542 million years ago) resulted in the divergence of major animal groups. Both metabolic transitions have allowed divergence of life forms on Earth, but evolution has not provided a way to prevent the onset of cancer. Since the entire history of humanity, with the exception in the last 100 years, the average lifespan was between 20 and 30 years; consequently, there might not be much evolutionary pressure to eradicate cancer as a disease of mostly elderly persons.

### 1.1. Somatic Mutation Theory vs. Metabolic Impairment Theory/Mitochondrial Theory of Cancer

At present, cancer is regarded a genetic disease arising from numerous mutations in oncogenes and tumor suppressor genes. Are gene mutations in the cell nucleus the causal event in the origin of cancer (as suggested by the somatic mutation theory) or is the damaged genetic material just the consequence and not the primary cause of cancer? Is cancer caused by damaged mitochondria (impaired mitochondrial function) and metabolic dysfunction, which activates the divergence of the glucose metabolism away from the energy production and stimulates cell growth (transition from oxidative phosphorylation to glycolysis/fermentation)? Is it genomic instability or debilitated energy metabolism that is essentially in charge of the cause of cancer? While tumor growth could be explained by the classical multistage model of carcinogenesis, the model does not provide rationale for the beginning of tumor development [22]. In the last 50 years, it has been accepted that initiation is the one event during which one or more mutations transform a normal somatic cell into a latent neoplastic cell, that is, a tumor cell still lacking multiplicative autonomy. This phase is then followed by promotion in which further mutations and proliferative stimuli induce the initiated cell to give rise to the progeny constituting the tumor. However, it remains to be elucidated what is the effect and what is the cause of normal-to-tumor cell transformation. Cancer was primarily considered as a type of somatic genetic disease in accordance with Boveri's cancer theory [23, 24] where harm to a cell's nuclear DNA underlies the change of a normal cell into a cancer cell [25–27]. Indeed, multiple and heterogeneous mutations are found in cancer cells [22]. The question however remains whether DNA mutations are the initiating event causing cancer or are they merely necessary contributors to the progression of tumor after its initiation? Are we battling cancer from the right front considering the hypothesis that DNA mutations as drivers are not that significant in initiation of tumors? Can tumors arise with regular division and mutation rates? Namely, spontaneous mutations are of the order 10^−5^ [28]. Estimated probability of mutating five genes, such as both alleles of a particular tumor suppressor gene and an oncogene, is 10^−20^ [29]. Thus, in terms of genetic hits in one cell, it is difficult to explain cancer formation as a result of the acquirement of random genetic mutations.

On the other hand, Seyfried et al. [27] explain cancer as essentially a metabolic disease related to disturbances in energy production through respiration and fermentation. According to the metabolic impairment theory/mitochondrial theory of cancer [4, 27, 30–34], cancer can best be explained as a class/kind of mitochondrial disease. As indicated by Warburg's hypothesis, cancer cells emerge from normal body cells through steady and irreversible harm to their respiratory capacity. Just those body cells which are able to increase glycolysis during intermittent respiratory damage are viewed as fit for forming cancers [31, 32]. The gene theory of cancer suggests that dysfunctional mitochondria could be the resultative and not the causative factor of cancers. On the other hand, the metabolic impairment theory indicates the contrary. Abnormal energy metabolism characterises most tumor cells in all types of tissues [14]. It was further observed that genes for glycolysis are excessively expressed in the major part of cancers explored into [35, 36]. What is more, the cancer cell metabolism is regulated also by metabolic oncogenes and tumor suppressor genes (e.g., K-ras, p53, PI3K, Akt, and MYC) which have evolved to regulate the Warburg effect [37]. Several studies indicate that the structure and function of tumor mitochondria are not normal and as such not capable to generate the adequate levels of energy [38–47]. The mitochondrial structure is intimately related to mitochondrial function. Abnormalities in both the content and composition of mitochondria have been observed in different tumor tissues *in vivo*. On the contrary, in different human and animal tumor cells, when they are grown in the *in vitro* conditions, in contrast to structural defects, reduced numbers or the absence of mitochondria is commonly not observed [27]. Moreover, some researchers observed that in different tumor types, mitochondria and OXPHOS are normal. However, such results were noticed mainly from the *in vitro* studies measuring oxygen consumption rates in tumor cells [48–53]. Already half a century ago, Warburg suggested that oxygen consumption could be comparable in normal and tumor cells although ATP formation is significantly lesser in tumor cells. The fact that the oxygen consumption rate can be similar or even greater in cultured tumor cells than in nontumorigenic cells was claimed also by different other authors [40, 54, 55]. However, it has been established that the oxygen consumption rate alone cannot be considered as an indicator of coupled respiration. This can be explained by the fact that some tumor cells consume oxygen while the glycolytically derived ATP is imported and hydrolysed through the mitochondrial adenine nucleotide transporter 2 so as to preserve the proton motive gradient [56]. Moreover, the cultured cell lines are usually derived from only a single cell or a few cells of a heterogeneous tumor. It can be concluded that mitochondria might appear functionally normal in many types of cultured tumor cells but appear structurally abnormal when evaluated in the tumor cells of many primary malignant cancers.

### 1.2. Mitochondrial Substrate-Level Phosphorylation (mSLP) Provides Energy Source for Cancer Cells: The Missing Link in Warburg's Theory

Reduced ATP formation through impaired oxidative phosphorylation or hypoxia must be compensated by tumor cells with an alternative source of energy. Glucose and glutamine represent available fermentable fuels, since acetate and branched chain amino acids are not present in adequate quantities and other amino acids can be used only with the presence of high-energy phosphates for the metabolic conversion to succinyl-CoA, which is the substrate for mSLP [57]. mSLP produces high-energy phosphates through glutaminolysis and represents a compensatory energy mechanism for cancer cells with insufficient or defective OXPHOS [58]. According to Seyfried et al. [57, 58], the missing link in Warburg's theory is the succinic acid fermentation which uses glutamine as a major substrate through sequential conversion of glutamine → glutamate → alpha‐ketoglutarate → succinyl‐CoA → succinate.

### 1.3. Deficiency of Energy: From Respiration to Fermentation

In order to enable multicellular life, cells must adapt to strict control of cell division and differentiation. Such cooperation works until there is enough energy supply in the form of NAD+ and ATP. However, both NAD+ levels and energy production in the form of ATP decline with age [59–61], and the incidence of many types of cancer increases with aging [62, 63].

**Figure 1:**
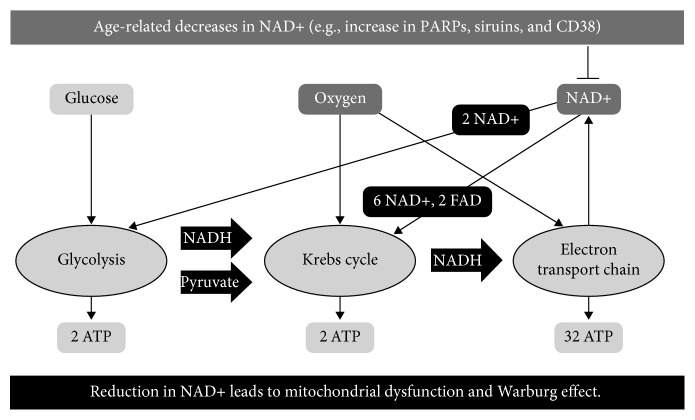
O_2_ and NAD+ as limiting factors in driving oxidative phosphorylation. The figure presents a hypothesis that in situations with limited availability of NAD+, the cells will activate the program which switches off Krebs cycle and electron transport chain (process consumes 6 NAD+) and favors glycolysis (process consumes 2 NAD+) in order to obtain energy, preserve NAD+, and avoid cell death through reduced ATP production and activation of apoptosis. ^∗^Abbreviations: PARP: poly(adenosine diphosphate [ADP] ribose) polymerases; CD38: NAD+ glycohydrolases; sirtuins: NAD-dependent histone deacetylase (“HDAC”) enzymes.

Age-related decline of NAD+ leads to mitochondrial dysfunction (Figure 1), which leads to the Warburg effect [64]. NAD+ or NAD+/NADH ratio can have an impact on the frequency of DNA mutation, epigenetic changes in DNA, and also metabolic programming [65]. The role of NAD+ is in accepting hydride equivalents, from glycolytic and TCA cycle metabolites, to form reduced NADH, which enables mitochondrial electron transport chain (ETC) to fuel oxidative phosphorylation [66]. In addition, high NAD+ levels regulate SIRT activity which influences metabolism, DNA repair, stress resistance, cell survival, inflammation, mitochondrial function, and lipid and glucose homeostasis, by targeting FOXO, PGC-1*α*, p53, NF-*κ*B, HIF-1*α*, and many other cellular targets [65].

According to Warburg's theory of cancer, the energy through fermentation gradually compensates for insufficient respiration [31, 67] which allows a cell to stay alive. NAD+ content is a basic protective factor at the beginning of carcinogenesis, and decreased NAD+ intracellular concentration might play a significant role in the process of cancer development by limiting energy production which negatively affects genomic stability by altering responses to stress and efficiency of the DNA repair [65, 68].

### 1.4. Potential Protumorigenic Side Effects of Increased NAD+

NAD+ can act as both pro- and antitumorigenic due to its mediated reactions on the mechanism of apoptotic cell death and inflammation. Different inflammatory soluble molecules secreted by senescent cells that could promote tumor growth and progression as well as NAD+ metabolism might influence the senescence-associated secretory phenotype (SASP) as discussed in the recent paper of Nacarelli et al. [69]. In their research, it was shown that increased NAD+ influences the inflammatory signaling of senescent cells in vivo in mouse models of pancreatic and ovarian cancers through the higher HMGAs and nicotinamide phosphoribosyltransferase (NAMPT) expression, which promotes the proinflammatory SASP through NAD+-mediated suppression of AMPK kinase, leading to suppression of the p53-mediated inhibition of p38 MAPK and enhanced NF-*κ*B activity [69]. Moreover, FK866, a compound which inhibits nicotinamide-recycling enzyme NAMPT/PBEF, which is the bottleneck for NAD biosynthesis, resulted in anticancer effect [70] as a tumor apoptosis inducer due to NAD+ depletion [71].

It seems that NAD+ levels are a critical protective factor in early carcinogenesis and might become a detrimental factor later in the cancer progression and promotion phase. Namely, during cancer promotion, progression and treatment-increased NAD+ levels could have deleterious effects on the malignancy process due to increased cell survival, growth advantage, increased resistance to radio and chemotherapy, and promotion of inflammation (reviewed in [65]). The tumor promoting vs. inhibiting properties of NAD+ depend on the stages of cancer development and NAD+ concentration/time-dependent activation of PARPs and sirtuins, which interfere with the cell survival. Sirtuins and PARPs could have both procancer and anticancer effects, and their role in cancer prevention and promotion remains to be fully elucidated [72–77].

### 1.5. Cancer and Mitochondrial Damage

There are many environmental agents (e.g., radiation, pollutants, and hypoxia) that humans are exposed to through their lives which damage mitochondria and cellular respiration through increased generation of reactive oxygen species (ROS). Therefore, ROS-induced damage to the respiratory system promotes a hypoxic-like state [31], stabilizes the transcription factor HIF, and upregulates glucose transporters into the cell. Additionally, oncogenes have to turn on because they are the transcription factors that upregulate the transporters for glucose and glutamine. The efficiency of mitochondrial oxidative phosphorylation decreases with age, and pseudohypoxia increases which leads to increased apoptosis (every day, 50-70 billion cells of a human body activate apoptotic death). However, in rare cases, a “renegade cell” decides not to sacrifice itself and undergo apoptotic cell death for higher purposes—to preserve the organism. Contrarily, in order to preserve its own life, a “selfish renegade cell” activates a prehistoric program in order to obtain enough energy levels. The aforementioned program activates fermentation and consequently shuts down genomic stability, tumor-suppressive control mechanisms, and mitochondrial apoptotic response [78] allowing such a cell to enter its primitive state. Activation of such processes results in a higher entropy state level inside the cell. A typical cell is a highly ordered low entropy system and invests much energy to keep the entropy of the system low. So as to keep up stable entropy, which is far from thermodynamic balance, living systems use information and energy. Energy loss due to impaired mitochondria limits supply of energy invested for damage repair, and genomic stability increases entropy and impairs order of the cell organization. Namely, glycolysis generates only two moles of ATP per one mol of glucose whereas oxidative phosphorylation generates about 36 mols of ATP per mol of glucose [79] (Figure 1). Hence, carcinogenesis represents a reverse process with the progressive functional decline, disordered morphology, and accumulation of mutations. Energy restriction due to mitochondrial dysfunction might represent the metabolic initiator that “triggers the genetic mutations that drive the somatic evolution of the malignant phenotype” [80].

In cases of glucose deprivation, efficient glucose consumption and catabolism are critical for survival. It was observed that cells switch to glycolysis in combination with lactate dehydrogenase as an adaptation to limited glucose availability [81]. When NAD+ levels within the cell become critically limited, both the TCA cycle in the mitochondria and glycolysis in the cytoplasm can be halted. Despite having an excess of available glucose, this can lead to cell death [82–85]. A less severe reduction in NAD+ levels (e.g., from 30 to 85%) has been observed in the muscle tissue of aged mice with an associated deterioration in mitochondrial function but not glycolysis [6, 64, 86–88]. It seems that cytoplasmic NAD+ pool is less susceptible to scarcity since “cytoplasmic NAD/NADH ratios range between 60 and 700 in a typical eukaryotic cell, while mitochondrial NAD/NADH ratios are maintained at 7 to 8” [89, 90]. The availability of NAD+ is thus critical for mitochondrial function [91–93].

### 1.6. Is the “Default” Metabolic Program Incorporated in the Cells of Multicellular Organisms' Glycolysis or Oxidative Phosphorylation?

It seems that cancer does not develop as a result of hypoxia due to damaged mitochondria or cell mass growth (hypoxic regions of tumors) that leads to impaired aerobic respiration as was first hypothesized by Warburg [31]. Some studies suggest that mitochondria are not damaged in some cancer cells [94–96], as discussed in the previous paragraph, and cancer cells seem to use glycolytic metabolism prior to the exposure to hypoxic conditions [97] as observed in leukemic cells [50, 98] and lung tumors which use aerobic glycolysis even though these tumor cells are exposed to high oxygen levels during tumorigenesis [99, 100]. Alteration in the metabolic switch to the aerobic glycolysis by cancer cells may thus result in the prehistoric (re)program that reverses premalignant cells to an embryonic program that supports cell growth by nutrient acquisition and metabolism. Before oxygen was formed in the atmosphere, proliferation and fermentation was the dominant phenotype and the default state of metazoan cells [101]. According to Szent-Györgyi [101], cancer is a condition of unrestricted cell development, which is typical of free-living cells 500,000 million years ago, before the existence of multicellular life. Cancer is a normal growth from before half a billion years ago, preceding the Cambrian time frame. That was before plants and before oxygen-rich atmosphere; life was just fermentation, with boundless telomerase. When nutrients are available, the unicellular organisms have evolutionary pressure to multiply as soon as possible by fermentation of glucose to generate biomass, which enables them to maintain the building blocks needed to produce a new cell [97]. In 1940, the French biologist Jacques Monod was the first to discover that genes can be regulated by metabolic readjustment in the experiment with *E. coli* fed on glucose or lactose sugar [102]. Although anaerobic glycolysis is less efficient, it is much more rapid than oxidative phosphorylation. Warburg observed that in the same amount of time a normal cell takes to consume one glucose molecule through OXPHOS, the cancer cell consumes 13 glucose molecules, only one of which through OXPHOS [103]. Such a switch can be explained from the evolutionary viewpoint as this may have helped a unicellular organism to speedily monopolize sugars when available and create an unfavorable environment for competing microorganisms [104].

### 1.7. Why Do Cancer Cells Prefer a Relatively Inefficient Way (in terms of ATP Production) of Extracting Energy from Glucose?

Warburg effect enables cancer cells to convert nutrients into building blocks to form different macromolecules in order to divide fast. Cancer cells must be directed either to cell death or to adaptation to a glycolytic phenotype once their cells reach the oxygen diffusion limit and become hypoxic. If “renegade cells” do not shift to such a primitive form of energy, they will die from apoptosis or lack of ATP. Therefore, cells deficient in ATP often undergo apoptosis [105]. Contrary, by activating glycolysis, “a renegade cell” stimulates cell division and suppresses apoptosis and differentiation [14] as well as the “multiunit teamwork.” Such a cell evolved to survive on its own. When cooperation is stopped, and fermentation is preferred, differentiation and specialization are reversed to a more primitive form, and transition to dedifferentiated cells is favored. Such a cell passes the energy needed for self-preservation/regeneration to increased reproduction; consequently, energy for the repair of cells and also the adaptive response to stress as cell cycle arrest regulation and apoptotic removal of damaged cells is depleted. Furthermore, glycolysis significantly diminishes cellular oxidative stress [106]. Both glucose and glutamine-derived glutamate are needed for synthesis of glutathione, which provides high antioxidant capacity and protect cancer cells from elevated ROS formation during chemo- and radiotherapies [107–109].

### 1.8. Why Did Evolution Preserve the Ability of Cells to Activate Aerobic Glycolysis?

The antagonistic pleiotropy hypothesis would explain fermentation as a beneficial process to the organism's fitness at the first week of embryo life when fast-growing cells of an embryo resemble more a cancer mass than normal differentiated tissue. An embryo must survive the first days without blood supply and oxygen. When the ovum reaches the uterus, it develops into a blastocyst consisting of over 100 cells. Upon entering the uterus, the embryo attaches into the uterine lining. Only after the embryo reaches the womb does it obtain blood supply and oxygen, which enables its organized growth [110]. However, later in life, the ability to activate “cancer genes” to drive glycolysis could become detrimental to the organism's fitness as a cell might become cancerous.

**Figure 2:**
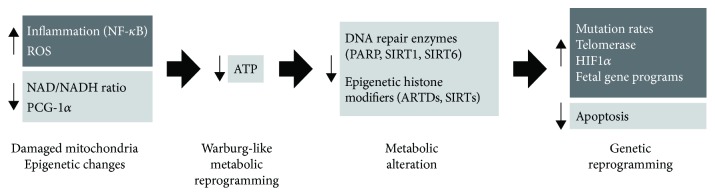
Metabolic alterations produce genetic alterations (activation of oncogenes and repression of tumor suppressor genes) which influence cancer development. What the causes are of metabolic alteration is still a matter of debate. Potential candidates involved in the metabolic switch from respiration to fermentation are increased inflammation, increased ROS formation, overstimulation of PARPs, decreased intracellular energy levels, and damaged respiration.

What is more, anaerobic glycolysis is activated during short, intense exercise, providing energy to escape during fight-or-flight response. After only 10-30 seconds of short-duration high-intensity anaerobic exercise, the majority of cellular energy come from the anaerobic glycolytic system manifested in the elevation of the blood-lactate level. This system provides ATP for up to 2–3 minutes. Then, the generation of energy switches back to oxidative phosphorylation [111–113]. While the acute switch from oxidative phosphorylation to anaerobic glycolysis is triggered by high-intensity anaerobic training, the cause of the permanent switch to chronic glycolysis remains unknown (Figure 2). One trigger might be increased and permanent inflammation and oxidative stress, which stabilize HIF-1-alpha, which advances a hypoxic-like (Warburg effect) state in the cell resulting in metabolic reinventing toward glycolysis and thus encouraging tumor development [114–116]. Anaerobic glycolysis and imperfect respiratory chain produce a lot of ROS and frame an endless loop which creates significantly more damage to mtDNA and decreases energy formation from oxidative phosphorylation and further invigorates fermentation.

### 1.9. CSC Metabolic Reprogramming

The cancer stem cell (CSC) hypothesis states that malignant tumors are initiated and maintained by a population of tumor cells that share similar biologic properties to normal adult stem cells [117]. Transformation of a normal stem cell into a CSC may occur through dysregulation of the proliferation and differentiation pathways or by inducing oncoprotein activity [118]. An alternative is the potential dedifferentiation of mutated cells so that these cells acquire stem cell-like characteristics [119], which is applicable to cells of all origins. It was observed that non-CSCs could be shifted to CSCs and vice versa in response to intrinsic and/or microenvironmental signals (e.g., oncogenes, tumor suppressor genes, hypoxia, oxidative stress, nutrient starvation, and epigenetics), which means that metabolic reprogramming might play a significant role during CSC transition [37]. Menendez et al. [37] argue that CSC bioenergetics might be another cancer and that metabolic reprogramming of CSCs has cancer-causing action. Increased glycolytic activity observed in early embryonic cells and high proliferation and diffusion are similar (or being reactivated) in cancer stem cells, which resume a more primitive metabolic pattern of energy production [13]. Cancer stem cells express the same metabolic defect as seen in all types of cancer cells. Mitochondrial function, redox status, and ROS formation play an important role in differentiation, maintenance, and self-renewal of CSC^13^. As in cancer cells, the stimulation of aerobic glycolysis supports, while the blockade of glycolytic enzymes blunts cancer-like metabolic reprogramming, phenomena observed in Induced Pluripotent Stem Cells (iPSCs) [120–123]. Even in the absence of genetic alterations, the Warburg effect and inhibition of OXPHOS are triggered in iPSCs by two primum movens: downregulation of the expression of the catalytic subunit of the AMP-activated protein kinase (AMPK) [124] and H+-ATPase synthase-geared metabolism switch [125–127]. Increased glycolysis in the presence of O_2_ and impaired oxidative phosphorylation are observed in both embryonic cells and CSC and other tumor cells [128].

### 1.10. Reprogramming of the Glycolytic Metabolism and Oxidative Phosphorylation: Is the Trigger the Inflammatory Stresses?

Numerous studies indicate a strong link between chronic inflammation and cancer (reviewed in [129–138]). Although mechanisms of chronic inflammation are very complex and the precise role of increased inflammation and cancer remain largely unknown, a nuclear factor-*κ*B (NF-*κ*B), considered as the master activator of inflammation [139], and p53, the major tumor suppressor, play a pivotal role. Activation of the NF-kappaB system increases the apoptotic resistance, activates the chronic inflammatory response, and reduces the autophagic cleansing [140]. Besides, macrophages that secrete cytokines and growth factors are attracted by the inflammatory response which promotes tumor cell growth and metastasis [141].

Chronic inflammation and accumulation of oxidative stress during aging also lead to NAD+ depletion [142], resulting in loss of sirtuin and PARP activity. Chronic inflammation will result in increased ROS formation leading to a decrease in intracellular NAD+ and cell death via energy restriction as a result of DNA strand breaks and PARP activation [143]. For example, in the brain cells, increased PARP activity, which leads to decreased NAD+, has been shown to decrease ATP as well as cause cell death [144, 145]. In particular, DNA repair enzyme PARPs utilize a lot of intracellular NAD+ (100 molecules of NAD+ when activated by one DNA break) and are in this manner in rivalry with sirtuins for the constrained supply of NAD+. Deacetylation by SIRT1 reprograms inflammation and cancer [146]. Constrained accessibility of NAD+ and reduced expression of SIRT1 may sustain aberrant chromatin structure and functions. Subsequently, reduced cellular NAD+ limits the efficacy of sirtuins (SIRT1), possibly deacetylating tumor suppressor proteins such as p53 [147]. p53 differentially controls a cluster of its target genes, encompassing the arrest of cell cycle, autophagy, apoptosis, and senescence, to apply its function in the damage of DNA and suppression of tumors. Consequently, a depletion of p53 gives a growth advantage to tumor cells; for example, it empowers cell survival under constraining nutrient conditions [148]. Moreover, NAD+-dependent tankyrases (PARP-5a and PARP-5b), which control telomerase activity and telomere maintenance, may likewise impact the cancer-causing process [149].

SIRT1 likewise impacts inflammation and cancer by straightly deacetylating targets like p65, p53, and NF-*κ*B, which produce proinflammatory products. NAD+ levels steadily decline with age [129] due to loss of SIRT3 activity in mitochondria, loss of PARP activity, and increased levels of NADase CD38 during aging [86, 142]. Since NF-*κ*B regulates the CD38 expression [150], the increase in low-grade inflammation with age might be the reason for NAD+ decline. Consequently, cells with high levels of CD38 use less oxygen, have increased lactate, and have dysfunctional mitochondria [142]. During chronic inflammation, NAD+ levels and SIRT transcription and/or protein levels are persistently reduced in different tissues [151]. Chronic inflammation and the release of proinflammatory mediators might thus reprogram cellular metabolism and energy production. For example, the induction of anabolic glycolysis is observed in cells of the immune system (e.g., monocytes and macrophages) exposed to inflammatory stress [152–155]. With increased age, the innate immune system does not efficiently clears the senescent cells as emitters of signals that drive inflammation and the vicious cycle initiates [156].

### 1.11. Prevention of Glycolysis and Reactivation of Mitochondrial Oxidative Metabolism: Approaches That Target Cell Energy Metabolism and Restore Mitochondrial Function

#### 1.11.1. Targeting Aerobic Glycolysis Pathways and the Warburg Effect

Many compounds affect aerobic glycolysis and would be efficient in depleting ATP in cells with mitochondrial defects and triggering cell death. Different small molecules target aerobic glycolysis and could be used as novel tumor therapeutics, for example, 2-deoxyglucose [157], lonidamine, 3-bromopyruvate [158, 159], imatinib, oxythiamine, and 6-aminonicotinamide [160–162]. Another way of action is to inhibit glucose transport by phloretin [163] or stimulation of mitochondrial oxidative metabolism through overexpression of mitochondrial frataxin, which inhibits tumor growth [164, 165]. Already in clinical use are imatinib and trastuzumab (Herceptin), which target signaling pathways linked to glucose metabolism [98, 166], primarily in those individuals with mutations in specific receptors linked to the insulin-like growth factor 1-Akt/protein kinase B (IGF-1/PI3K/Akt) pathway. Many studies are showing that the Warburg effect can be targeted with dichloroacetate (DCA) and increased mitochondrial activity of glutaminolysis with arsenic trioxide (ATO). It was observed that DCA induces apoptosis in cancer cells but does not induce apoptosis in normal cells [167–172].

There are many agents that can act as anti-Warburg agents. Their way of acting is to increase the NAD+ levels and promote the oxidative metabolism [173]. For example, SIRT3 can restrain the “Warburg effect” by controlling HIF-1*α* and change the cancer cell metabolism programming from highly glycolytic toward oxidative phosphorylation [116, 174, 175]. Besides, by inactivating HIF-1*α*, SIRT1 represses HIF-1 target genes and adversely effects tumor growth and angiogenesis [176]. By increasing levels of sirtuins, PARPs, and PGC-1*α*, oxidative metabolism, inflammation, epigenetic gene silencing, cell cycle control, genome stability, apoptosis, stress resistance, energy efficiency, DNA repair, cell death, genome integrity, cellular differentiation, gene expression, and antiaging could be promoted.

Finally, mitochondria could be used as a potential anticancer drug target. The apoptotic process could be regulated by reactivating or by transferring mitochondria [5].

#### 1.11.2. Enhancing Mitochondrial Biogenesis and Efficacy and Boosting Oxidative Metabolism

By enhancing the bioavailability of NAD+, oxidative capacity of mitochondria could be restored. NAD+ levels could be raised with exercise, restriction of calories (CR), and ingestion of NAD+ precursors and intermediates. Alternatively, NAD+ bioavailability can be increased by using poly-ADP-ribose polymerase (PARP), CD 38, and SAM1 inhibitors [60, 65, 177–185]. Consequently, increased NAD+ levels could activate PARPs and sirtuins which control the genes that play a role in the process of DNA repair and maintenance [173]. Additionally, different NAD(+) precursors can be used through distinct metabolic pathways to form NAD(+), such as nicotinamide, nicotinamide mononucleotide, tryptophan, nicotinic acid, and nicotinamide riboside. Further, consumption of foods that contain molecules necessary for respiratory enzyme function (riboflavin, nicotinamide, iron salts, and pantothenic acid) could help to maintain health when it is combined with dietary energy restriction [186] since CR increases the efficiency of the electron transport in the mitochondrial respiratory chain [187]. Pyrroloquinoline quinone (PQQ) might increase the number and efficiency of mitochondria. PQQ interacts with cell signaling pathways and influences energy-related mitochondrial metabolism [188]. The mitochondrial biogenesis is stimulated through a pathway that activates the cAMP response element-binding protein (CREB) and peroxisome proliferator-activated receptor gamma coactivator-1alpha (PGC-1alpha) [189].

While raising NAD+ levels for cancer prevention might be beneficial, increasing NAD+ levels might be detrimental during the precancerous stage or once the cancer is formed [65] (discussed in the previous paragraph).

#### 1.11.3. Increasing the Intracellular Oxygen Level with Hyperbaric Oxygen Therapy

Hyperbaric oxygen therapy raises oxygen levels in tumors and reverses the cancer-promoting effects of tumor hypoxia [190, 191]. By enhancing oxygen delivery to cells, more ATP can be obtained through oxidative phosphorylation since cells make use of oxygen acting as a final electron acceptor in the process of generating ATP in their mitochondria and mitochondrial integrity could be preserved [192]. Poff et al. [193] observed that a combination of the ketogenic diet with hyperbaric oxygen therapy resulted in a noticeable drop in blood sugar and the rate of tumor development and increased mean survival of mice with systemic metastatic cancer.

#### 1.11.4. Increasing Regulation of Contact Inhibition (Density-Dependent Inhibition) and Proliferation

Due to the loss of growth control, the growth and division of cells are uncontrolled. Cells should be informed that they are a part of a multicellular organism and that they have to obey the control of proliferation or to activate apoptosis if being damaged. This could be achieved by increasing the response to the signals that cause healthy cells to cease proliferation and enter the G0 phase and by decreasing the production of growth factors that stimulate cancer cells to own proliferation [194–196].

#### 1.11.5. Targeting Glucose and Elevating Blood Ketone Bodies through a Calorie-Restricted Ketogenic Diet (KD-R)

The energy metabolism in glycolysis-dependent tumors can be targeted by a transition from carbohydrate to ketones. Healthy cells can be protected from such glycolytic inhibition, and the brain can be protected from hypoglycaemia by elevating blood ketones, which occurs when a low-carbohydrate and a high-fat ketogenic diet is carried out in limited amounts [34]. Exogenous ketone supplementation on its own inhibits cancer cell proliferation and viability *in vitro*, slows tumor growth, and prolongs survival *in vivo* [197]. Caloric restriction/KD-R reduces carbons needed in glycolytic and pentose phosphate pathways in order to provide ATP, precursors for lipid and nucleotide synthesis and formation of antioxidant glutathione. Due to impaired mitochondrial function, cancer cells are depending on substrate-level phosphorylation, and during ketone body metabolism, mSLP is bypassed. Ketone bodies may elicit their anticancer effects, most likely by glycolytic enzyme inhibition [198]. Numerous research studies documented that *in vitro* cancer cells were deficient in metabolizing of ketone bodies [199, 200]. Ketone bodies generate ATP energy only through oxidative respiration in the mitochondria and cannot be fermented. While dietary energy reduction lowers blood glucose levels and restricts the energy supply to cancer cells, some of the tumor cells might still obtain enough energy to survive due to the endogenous glucose and amino acid influx.

#### 1.11.6. Targeting Glutamine

For cell growth and division, cells need a supply of carbon, nitrogen, free energy, and reducing equivalents, which can be obtained through glucose and glutamine metabolism [97]. Glutamine functions as a significant energy metabolite for some cancers. For example, tumors with deregulated MYC expression may be less sensitive to inhibition of glycolysis than tumors with overactivation of the Akt pathway [178, 201]. Glutamine provides a source of carbon and nitrogen needed for nucleotide synthesis, and targeting glutamine metabolism with the glutamine antagonist 6-diazo-5-oxo-norleucine (DON) might be used in cancer treatment [202–204]. For example, it was shown in two glioblastoma mouse models that administration of DON and calorically restricted ketogenic (KD-R) diet killed tumor cells, reversing disease symptoms and increasing overall mouse survival. Simultaneous administration of DON and KD-R both targeted substrate-level phosphorylation reactions in mitochondria (glutaminolysis) and in the cytoplasm (glycolysis), respectively, thus enabling ATP formation and synthesis of proteins, nucleotides, and lipids [205]. It should be stressed that glutamine is needed for appropriate functioning of the immune system and the gut [206]; thus, glutamine targeting is more demanding than glucose targeting.

#### 1.11.7. “Press-Pulse” Therapeutic Strategy

The team of Seyfried developed a so-called “press-pulse” therapeutic strategy [107, 207]. The general concept of press disturbances (chronic metabolic stress on tumor cell energy disturbance) and pulse disturbances (acute metabolic stressors that restrict glucose and glutamine availability) could be applied for the management of cancer. Press therapies reduce systemic glucose concentrations and elevate ketone bodies; pulse therapies use cocktails which interfere with glycolysis and glutaminolysis metabolic pathways [107, 207].

#### 1.11.8. Targeting Insulin/Insulin-Like Growth Factor (IGF) Signaling, Mammalian Target of Rapamycin (mTOR), and AMP-Activated Protein Kinase (AMPK) Pathways

Metabolic therapies that lower circulating glucose levels were reported to significantly reduce growth and progression of numerous tumor types [14]. A number of epidemiological studies initially concluded that in patients with diabetes who controlled their blood sugar levels by taking metformin, the development of cancer was less likely. It was also observed that their survival rate was improved once cancer was initiated. Several retrospective studies indicated that people with diabetes had increased cancer mortality compared with nondiabetics and that people with diabetes on metformin had a substantially (∼40%) reduced cancer burden compared with diabetics on other treatments [208]. For example, glucose reduction lowers insulin and IGF-1 levels, which is required for driving tumor cell metabolism and growth [209, 210]. Caloric restriction specifically influences the IGF-1/PI3K/Akt/HIF-1*α* signaling pathway, which regulates several cancer hallmarks like evasion of apoptosis, cell proliferation, and angiogenesis [14]. Diabetes drugs metformin and phenformin might have benefit in cancer prevention as activators of AMPK in cells. AMPK is activated by also salicylate in vitro and by “nutraceuticals” such as resveratrol, epigallocatechin gallate, and berberine, which activate AMPK by inhibiting mitochondrial ATP production [211]. AMPK is also activated in the resting muscle with 5-aminoimidazole-4-carboxamide-riboside (AICAR), which enters the muscle and is phosphorylated to ZMP (5-aminoimidazole-4-carboxamide-1-*β*-D-ribofuranosyl-5′-monophosphate, an AMP analog). ZMP is a nucleotide that mimics the effect of 5′-AMP [212–214]. 3,3′-Diindolylmethane (DIM) from cruciferous vegetables and epigallocatechin gallate (EGCG) from green tea have been reported to be effective AMPK activators in a prostate or breast cancer model system, both *in vitro* and *in vivo* [215]. Additionally, AMPK was demonstrated to suppress tumor growth in vivo as a negative regulator of the Warburg effect [216]. AMPK in muscles is activated, in response to both *in vivo* exercise and *ex vivo* contraction [217, 218]. The varied role of AMPK on cancer cell survival and tumor progression and suppression is explained in detail elsewhere [219]. The induction of AMPK activity inhibits the activity of rapamycin (mTOR) [220]. Mammalian target of rapamycin (mTOR) regulates a translational control over cell division, growth, and energy metabolism, while IGF-1/Akt regulates the transcriptional regulators of these processes. The inhibition of apoptosis and the promotion of growth and division are, therefore, the result of the activated IGF/Akt pathway [221]. A serine/threonine protein kinase mTOR controls the growth, proliferation, motility, and survival of cells; protein synthesis; and transcription [222, 223] in response to nutrients (e.g., glucose and amino acids), growth factors (e.g., increased levels of insulin, IGF-1, and platelet-derived growth factor (PDGF)), and cellular energy status (ATP). CR and p53 (a nuclear transcription factor with a proapoptotic function) may also inhibit mTOR activity [148].

#### 1.11.9. Shifting from Anabolic to Catabolic Metabolism Suppresses High Rates of Proliferation

Anabolic pathways that advance growth are stimulated in cancer by means of tumorigenic mutations, especially PI3K-mTOR signaling [224]. PI3K-Akt-mTOR network signaling, where many oncogenes and tumor suppressors reside, is acquired with minimal reliance on external stimulation by growth factors [225]. Additionally, glucose metabolism generates glycolytic intermediates (hexosamine pathway, PPP, and one-carbon metabolism) which promote anabolic pathways that support cell growth [226]. On the other hand, only a couple of short periods of fasting activates AMPK, which triggers repair and catabolic processes. Alongside, AMPK-mediated inhibition of mTOR activity [226] and downstream anabolic pathways establishes separation of anabolic and catabolic processes [227]. Tumor cells have aberrant activation of mTORC1 that evokes an anabolism leading to nucleotide, protein, and lipid synthesis. A depletion of tumor suppressors, such as p53, or activation of oncogenes, e.g., MYC, to a greater extent enhances an anabolic growth program by metabolic gene transcriptional regulation.

Currently, there are many other strategies under investigation targeting mitochondrial energy metabolism to 2inhibit or delay tumor growth. Some of them deal with DNA methylation pattern, epigenetic reprogramming, and aberrant microRNA (miRNA) levels and/or investigate the role of intermediates of the Krebs cycle on “nonmetabolic” signaling which alters the immune system, the role of DJ-1 (Parkinsonism-associated deglycase) as a modulator of mitochondrial metabolic efficiency and a switch between glycolysis and oxidative phosphorylation, and the role of bouchardatine in suppressing cancer by disrupting its metabolic pathways via activating the SIRT1-PGC-1*α*-UCP2 axis. Detailed descriptions of their principles are beyond the scope of this paper. More information can be found elsewhere [228–231].

### 1.12. Chemoresistance

Drug-resistant tumor cells arise in a large part from the damage to respiration in bystander precancerous cells. While cytotoxic drugs and radiation create tumor cells that become highly resistant to the classical treatment approaches, this is not probable when dietary energy reduction and approaches aimed at reversing abnormal energy metabolism and growth behavior in tumor cells are used [107, 232]. Chemoresistance is the result of the fermentation metabolism in the tumor cells. Glucose and glutamine contribute to the synthesis of glutathione, which protects tumor cells from oxidative stress [205]. Inhibition of glycolysis in cancer cells increases the sensitivity to common anticancer agents and overcomes the drug resistance [232]. Dietary restriction, periodic fasting, and fasting-mimicking diets are emerging as interventions used to prevent and treat cancer in combination with chemo- and radiotherapy [233–235].

## 2. Conclusion

A clear understanding of the origins of cancer is the basis of successful strategies for effective cancer prevention and management. Results are indicating that the carcinogenic process is not driven by the accumulation of random or stochastic genetic mutations, but instead, a mitochondrial metabolic disease [4] was presented. However, it remains to be elucidated what exactly triggers the reprogrammed metabolism in cancer cells. Additional studies are needed to investigate the causation-consequence relationship between metabolic abnormalities and the causation of the genetic mutations and, on the other hand, the mutation ability to trigger the metabolic abnormalities.

Both metabolic and standard cytotoxicity-based treatment approaches should be coupled. Strategies that restore mitochondrial metabolism/functions could have both tumor preventive (e.g., caloric restriction or intermittent fasting) and therapeutic implications in cancer (use of drugs, such as glutamine antagonist and 6-diazo-5-oxo-L-norleucine (DON), and others including KD-R). Evidence was presented that restoring redox homeostasis and reactivation of mitochondrial oxidative metabolism are important factors in cancer prevention. Preclinical studies are needed, followed by controlled-randomized clinical trials, investigating strategies to restore mitochondrial metabolism as well as synergistic effect of metabolic and standard cytotoxicity-based treatment approaches. Without findings of additional studies, no specific therapy can be currently favorited. The efficacy of the proposed treatment approaches should be further studied to determine their potential for clinical use in the future.
